# Construction of a Turn Off-On-Off Fluorescent System Based on Competitive Coordination of Cu^2+^ between 6,7-Dihydroxycoumarin and Pyrophosphate Ion for Sensitive Assay of Pyrophosphatase Activity

**DOI:** 10.1155/2016/4306838

**Published:** 2016-09-27

**Authors:** Lingzhi Zhao, Liu Zhao, Yanqing Miao, Chunye Liu, Chenxiao Zhang

**Affiliations:** ^1^Department of Pharmacy, Xi'an Medical College, Xi'an 710021, China; ^2^Laboratory of Analytical Chemistry for Life Science of Shaanxi Province, School of Chemistry and Chemical Engineering, Shaanxi Normal University, Xi'an 710062, China; ^3^Beijing Research Center of Agricultural Standards and Testing, Beijing 100097, China

## Abstract

The detection of pyrophosphatase (PPase) activity is of great significance in diagnosing diseases and understanding the function of PPase-related biological events. This study constructed a turn off-on-off fluorescent system for PPase activity assay based on PPase-regulated competitive coordination of Cu^2+^ between a water-soluble fluorescent probe 6,7-dihydroxycoumarin (DHC) and pyrophosphate (PPi). The probe DHC can coordinate with Cu^2+^ and consequently display on-off type fluorescence response. Furthermore, the in situ formed nonfluorescent Cu^2+^-DHC complex can act as an effective off-on type fluorescent probe for sensing PPi due to the higher coordination reactivity between Cu^2+^ and PPi than that between Cu^2+^ and DHC. The subsequent addition of PPase to the mixture containing Cu^2+^, DHC, and PPi leads to the fluorescence requenching of the system again (an off state) because PPase catalyzes the hydrolysis of PPi into orthophosphate in the reaction system. Under the optimum conditions, the decrease of the fluorescence intensity of DHC-Cu^2+^-PPi system was linear with the increase of the PPase activity in the range from 0.1 to 0.3 U. The detection limit was down to 0.028 U PPase (*S*/*N* = 3). Moreover, the as-established system was also applied to evaluate PPase inhibitor. This study offers a simple yet effective method for the detection of PPase activity.

## 1. Introduction

As one kind of the most important biological anions, pyrophosphate (P_2_O_7_^4−^, PPi) is one of the by-products of adenosine triphosphate (ATP) hydrolysis and can be used as a potential biomarker for the clinic diagnosis and therapy of familial chondrocalcinosis or calcium pyrophosphate crystal deposition disease [1–4]. Inorganic pyrophosphatase (PPase) is a kind of ubiquitous enzyme that specially hydrolyzes pyrophosphate (PPi) to orthophosphate (Pi), providing a thermodynamic pull for many different biosynthetic reactions, and has been demonstrated to be directly relevant to phosphorus metabolism, carbohydrate metabolism, and evolutionary events [5–7]. Moreover, many diseases, such as hyperthyroidism, colorectal cancer, and lung adenocarcinomas, are associated with the expression level and the abnormal regulation of PPase [8, 9]. Therefore, there is an urgent demand for developing technically simple yet effective methods for real-time PPase activity monitoring. Up to date, various analytical methods and techniques have been developed for PPase activity assay, such as radioactive label-based chromatography, colorimetry assay, and fluorescence methods a [10–16]. Most of these methods are based on monitoring the change in the amount of the PPi substrate or Pi product. For instance, Deng et al. [13] demonstrated a new colorimetric method for real-time PPase activity assay based on reversible tuning of the dispersion/aggregation states of gold nanoparticles (Au-NPs) by controlling the coordination of Cu^2+^ between cysteine and PPi with PPase. Zhang et al. [14] developed a convenient colorimetric strategy for PPase activity detection based on an inhibition effect of PPi on the Cu^2+^-catalyzed ABTS-H_2_O_2_ reaction. Therefore, the key to developing PPase detection methods is the development of effective methods for the sensing of the PPi substrate or Pi product.

For PPi detection, since PPi is usually chelated with metal ions to form coordination complexes, hence, metal complex-based chemosensors based on metal ion-ligand coordination interaction are highly interesting to researchers [17–27]. This strategy is based on the metal ion displacement of the metal complex by PPi due to the stronger coordination interaction between metal ion and PPi. Lin et al. [24] presented an electrochemical method for the determination of PPi that is based on the competitive coordination of Cu^2+^ between a nanofilm of cysteine and dissolved PPi. It was reported that the stability constant (*K*) of the complex formed by Cu^2+^ and PPi is log⁡*K*_Cu PPi_ = 12.45 [28], suggesting that the coordination ability of PPi with Cu^2+^ is stronger than that PPi with other divalent metal ions. Therefore, in this study, with the purpose to simplify the assay process for manufacturing portable and affordable devices, we constructed a turn off-on-off fluorescent system in aqueous media on the basis of competitive coordination of Cu^2+^ between water-soluble organic dyes 6,7-dihydroxycoumarin (DHC) and PPi for quantitatively analyzing PPase activity.

**Figure 1:**
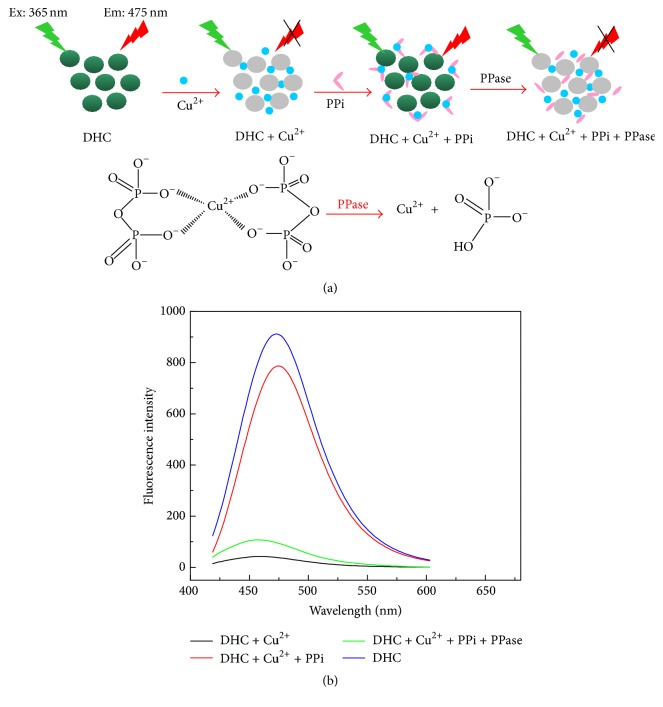
(a) Schematic illustration of the turn off-on-off fluorescent strategy based on competitive coordination of Cu^2+^ between DHC and PPi for PPase activity assay. (b) Fluorescence spectra of the mixtures prepared by separate addition of 500 *μ*M DHC; 500 *μ*M DHC and 200 *μ*M Cu^2+^; 500 *μ*M DHC and 200 *μ*M Cu^2+^ and 200 *μ*M PPi; 500 *μ*M DHC and 200 *μ*M Cu^2+^ and 200 *μ*M PPi and 0.25 U PPase. Buffer, HEPES buffer aqueous solution (10 mM, pH 6.5); the reaction temperature, 25°C; cuvette width: 1 mm. *λ*_ex_ = 375 nm.

Fluorescence methods possess innate advantages over other methods, such as designability of optical probes, the simplicity of implementation, high sensitivity, excellent selectivity, and fast response time. Herein, an effective fluorescence method for highly sensitive assay of PPase activity has been developed in the physiological buffer, which utilizes a simple competitive coordination reactivity of Cu^2+^ between DHC and PPi. As shown in Figure 1, initially, the strong fluorescence emission peak of DHC was observed at 475 nm. Upon the presence of Cu^2+^, the complex between Cu^2+^ and DHC was formed through the coordination interaction of the O atom in hydroxy groups of DHC and Cu^2+^, leading to the fluorescence quenching of DHC (an off state). Further addition of PPi to the dispersion of Cu^2+^-DHC complex enables the fluorescence emission intensity of DHC restored substantially (an on state) owing to the higher binding affinity between PPi and Cu^2+^ than that between DHC and Cu^2+^. On the other hand, the addition of PPase to the Cu^2+^-PPi complex containing DHC obviously leads to requenching the fluorescence (an off state), because PPase could catalyze the hydrolysis of PPi into Pi (herein, Pi must be HPO_4_^2−^ in pH 6.5), which cannot chelate Cu^2+^ to form the complex and thus Cu^2+^ was released. To the best of our knowledge, the as-designed turn off-on-off fluorescence sensing system is convenient without complicated protocols and exhibits excellent PPi sensing selectivity over other anions and structural analogues and excellent performances for sensitive assay of PPase activity in the physiological buffer compared to that of the existing methods, which paves a fluorescence route to the fast and simple clinic detection of PPi, the hydrolytic activity of PPases, and screening of PPase inhibitors.

## 2. Experimental

### 2.1. Materials and Instrumentation

6,7-Dihydroxycoumarin (DHC), adenosine 5′-triphosphate disodium salt hydrate (ATP), adenosine 5-diphosphate sodium salt (ADP), adenosine 5-monophosphate monohydrate (AMP), glutathione (GSH), L-cysteine (Cys), and homocysteine (Hcy) were purchased from Aladdin Company (China). Potassium pyrophosphate (PPi), Na_2_SO_4_, NaCl, KNO_3_, Na_2_SO_3_, Na_2_S_2_O_3_, NaH_2_PO_4_, Na_2_HPO_4_ (Pi), Na_3_PO_4_, Zn(NO_3_)_2_, Fe(NO_3_)_3_, Cu(NO_3_)_2_, CaCl_2_, MgCl_2_, NaF, and other inorganic salts were purchased from Sinopharm Chemical Reagent Co., Ltd. (China). Bovine serum albumin (BSA) was obtained from Shanghai Sangon Biological Engineering Technology and Services Co., Ltd. (China). Prostate specific antigen (PSA) was obtained from Fitzgerald Industries International, Inc. (USA). Human immunoglobulin G (IgG) was obtained from Beijing Middle Mountain Gold Bridge Biological Technology Co., Ltd. (China). Albumin chicken egg protein was obtained from Sino-American Biotechnology Co., Ltd. (China). Baker's yeast inorganic pyrophosphatase (PPase, EC 3.6.1.1) was purchased from Sigma (USA). One unit of Baker's yeast PPase liberates 1.0 *μ*mol of inorganic orthophosphate/min at pH 7.2 and 25°C. All other chemicals were at least analytical grade reagents and used without further purification. HEPES buffer aqueous solution (10 mM, pH 6.5) was prepared by adjusting commercial 10.0 mM HEPES to its pH of 6.5 using 2 M NaOH. All aqueous solutions were prepared with Milli-Q water (18.2 M Ω·cm^−1^). Unless otherwise pointed out, all experiments were carried out at room temperature.

The absorbance spectra and photoluminescence (PL) spectra were recorded on a UV-Vis spectrophotometer (UV-2450, Shimadzu Corporation, Japan) and a Cary Eclipse fluorescence spectrophotometer (Varian, USA), respectively.

### 2.2. UV-Vis and Fluorescence Experiments

The detection measurements were conducted as follows: first, the stock solution of each reactant was prepared by dissolving the compound in HEPES buffered aqueous solution (10 mM, pH 6.5). After that, the solution of DHC, Cu^2+^, PPi, or other reactants was sequentially added; the final volume is 500 *μ*L. The resulting mixture was then incubated in a 25°C water bath for 30 min. The UV-Vis and fluorescence spectra were recorded immediately. The present concentration of each reactant was the final concentration.

### 2.3. PPase Activity Assay

PPase activity assay was performed under the following procedures. A 5 *μ*L volume of PPase with different activities ranging from 0.1 to 1 U was added to the aqueous dispersion consisting of 500 *μ*M DHC, 200 *μ*M Cu^2+^, 200 *μ*M PPi, and 500 *μ*M Mg^2+^. Fluorescence spectra of the dispersions were consecutively recorded in the different time at 25°C. The final volume is 500 *μ*L.

### 2.4. Phosphate Assay for PPase Activity Based on Colorimetric Method of Molybdenum Blue [29]

The reaction was started by mixing 0.25 U PPase and the substrate PPi and activating agent Mg^2+^ in a 1.5 mL microfuge tube (the final concentration of PPi and Mg^2+^ is 500 *μ*M). In order to measure effects of Cu^2+^ and DHC on PPase activity, 500 *μ*M DHC or 200 *μ*M Cu^2+^ was added in the above-mentioned reactant solution. After 30 min, the reaction was stopped by adding 35 *μ*L of SDS-EDTA (13.3% SDS, 0.12 M EDTA, pH 6.5). And then, 265 *μ*L of color development reagent (0.5% Fe_2_SO_4_, 0.5% ammonium molybdate, and 0.5 M H_2_SO_4_) was added into the tube. The final volume is 400 *μ*L. Color was allowed to develop for 15 min and read in a microplate reader at 650 nm (EMax Plus Microplate Reader, Molecular devices, USA).

### 2.5. Inhibitor Efficiency Evaluation

For investigating the inhibition efficiency of NaF on PPase activity, 5 *μ*L of PPase (0.25 U) was initially added into 60 *μ*L of aqueous solutions of NaF with different concentrations (100 nM, 1 *μ*M, 5 *μ*M, 10 *μ*M, 50 *μ*M, 100 *μ*M, and 500 *μ*M) in the presence of 500 *μ*M Mg^2+^ in a 25°C water bath for 15 min. After 15 min, the NaF-treated PPase was added to the aqueous dispersion consisting of DHC (500 *μ*M), Cu^2+^ (200 *μ*M), and PPi (200 *μ*M). After 30 min, Fluorescence measurements of the dispersions were obtained. The final volume is 500 *μ*L.

## 3. Results and Discussion

### 3.1. Principle of Fluorometric Assay of Inorganic Pyrophosphatase Activity

To begin with, as displayed in Figure 1(a), the fluorescence spectrum of 6,7-dihydroxycoumarin (DHC) dispersion in HEPES buffer (10 mM, pH 6.5) was obtained by excitation of the fluorophore at 375 nm, and a strong emission peak was observed at 475 nm (an on state) (Figure 1(b), blue curve). Intriguingly, the addition of Cu^2+^ to this aqueous dispersion dramatically quenched the fluorescence of DHC through the coordination chemistry between Cu^2+^ and DHC (an off state) (Figure 1(b), black curve) and the formation of Cu^2+^-DHC complex. Further addition of PPi to the dispersion of Cu^2+^-DHC complex enabled the peak intensity of the emission spectrum of DHC restored substantially (an on state) (Figure 1(b), red curve) because of a stronger coordination between PPi and Cu^2+^ than that between DHC and Cu^2+^. On the other hand, the addition of PPase to the Cu^2+^-PPi complex containing DHC obviously leads to the fluorescence quenching again (an off state) (Figure 1(b), green curve) because Pi, which is produced from PPase-catalyzed hydrolysis of PPi, cannot chelate Cu^2+^ to form the complex. The large difference in the capability between PPi and other phosphate anions to compete with DHC in the coordination with Cu^2+^ will be investigated in subsequent fluorescence spectrum study, which actually forms the basis for construction of the turn off-on-off fluorescent system for sensitive assay of PPase activity as demonstrated in Figure 1.

### 3.2. The Feasibility of Fluorometric Assay

**Figure 2:**
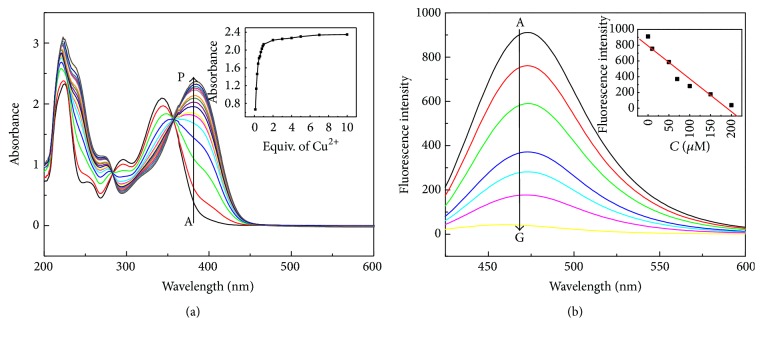
(a) UV-Vis absorption responses of DHC (500 *μ*M) upon the addition of different concentrations of Cu^2+^ (0, 0.1, 0.2, 0.3, 0.4, 0.5, 0.6, 0.7, 0.8, 0.9, 1.0, 2.0, 3.0, 4.0, 5.0, 7.0, and 10.0 equiv.) in HEPES buffer aqueous solution (10 mM, pH 6.5). Inset: the corresponding changes of absorbance of DHC at 375 nm in the presence of different concentrations of Cu^2+^. (b) Fluorescence titration of DHC (500 *μ*M) with Cu^2+^ (0, 10, 30, 50, 70, 100, and 200 *μ*M) in HEPES aqueous buffer (10 mM, pH 6.5). Inset shows fluorescence intensity changes of DHC at 475 nm as a function of the Cu^2+^ concentration. Other reaction conditions are the same as Figure 1.

At first, the interaction of DHC with Cu^2+^ was investigated by UV-Vis spectrophotometric titration in HEPES buffer (10 mM, pH 6.5). Initially, the UV-Vis absorption spectrum of DHC displayed a strong absorbance band centered at about 340 nm, as shown in Figure 2(a), which is the characteristic absorption profile of DHC. The addition of Cu^2+^ to this aqueous dispersion produced a new absorption peak at about 375 nm. This phenomenon was elucidated by the coordination chemistry between Cu^2+^ and DHC; the O atom in two hydroxy groups of DHC can coordinate with Cu^2+^ to form the Cu-DHC complex. Such a coordination interaction eventually leads to the red shift of the absorption peak of DHC. In order to judge the coordination number of Cu-DHC complex, Cu^2+^ titration experiment was performed. As shown in Figure 2(a), upon the addition of increasing amounts of Cu^2+^, a new absorption peak at 375 nm increased and the absorption peak of DHC at 340 nm gradually decreased in the concentration of introduced Cu^2+^ (0-1 equiv. of Cu^2+^). Appearance of an isosbestic point at 355 nm also demonstrated the formation of a well-defined complex between DHC and Cu^2+^. When the concentration of Cu^2+^ increased from 1 to 10 equiv. (Figure 2(a), inset), the absorbance band was no longer changed noticeably, suggesting 1 : 1 stoichiometric complexation of DHC with Cu^2+^.

DHC exhibited the maximum emission at a wavelength of 475 nm in HEPES buffer (10 mM, pH 6.5) under the optimal fluorescence excitation at a wavelength of 365 nm (Figure 2(b)). It is clearly seen that the distinct fluorescence peak of DHC decreased markedly in the presence of Cu^2+^. This fluorescence quenching might be attributed to the coordination interaction between Cu^2+^ and DHC and the aggregation of DHC after Cu^2+^ reacted with DHC. To quantitatively evaluate the fluorescence response of DHC toward Cu^2+^, fluorescence titration with Cu^2+^ in varying concentrations was conducted. As depicted in Figure 2(b), with the increasing concentration of Cu^2+^, the fluorescence emission intensity of DHC decreased gradually and was directly proportional to the concentrations of Cu^2+^ in the range from 10 to 200 *μ*M (*F* = 685.9 − 5.53*C*_Cu^2+^_, *R* ^2^ = 0.984). The fluorescence of DHC (500 *μ*M) was quenched by approximately 95% with the presence of 200 *μ*M Cu^2+^. Therefore, 200 *μ*M Cu^2+^ was chosen in subsequent experiments.

**Figure 3 fig3:**
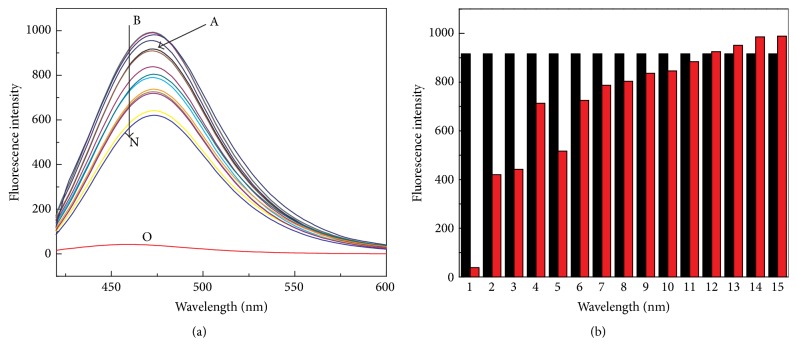
(a) Fluorescence changes of DHC (500 *μ*M) in the absence (curve A) and the presence of various cations (curves B to O) in HEPES buffer aqueous solution (10 mM, pH 6.5). (b) Fluorescence responses of DHC (500 *μ*M) at 475 nm in the absence (black bars) and presence of different cations (1 to 15: Cu^2+^, Fe^3+^, Fe^2+^, Zn^2+^, and Al^3+^ 200 *μ*M; Cr^3+^, Pb^2+^, Ni^2+^, Cd^3+^, Co^2+^, and Ag^+^, 100 *μ*M; Na^+^, K^+^, Mg^2+^, and Ca^2+^, 500 *μ*M) (red bars). Other reaction conditions are the same as Figure 1.

To gain insight into the sensing properties of DHC, the selectivity of DHC for sensing Cu^2+^ was evaluated by comparing its fluorescence response toward Cu^2+^ with those of 13 other kinds of conventional metal ions including Fe^3+^, Fe^2+^, Zn^2+^, Al^3+^, Cr^3+^, Ag^+^, Pb^2+^, Cd^3+^, Co^2+^, Mg^2+^, Na^+^, K^+^, and Ca^2+^. The emission characteristics were examined in the presence of various metal species. As shown in Figure 3(b), most cations caused minor fluorescence intensity changes (the red columns in Figure 3(b)), and the quenching efficiency for 200 *μ*M Cu^2+^ came to 95% while that for 6 other kinds of metal ions did not exceed 15%. 200 *μ*M Fe^3+^ and Fe^2+^ caused the largest fluorescence intensity quenching and the quenching efficiency reached about 50%. But in the physiological conditions, such as in human serum, the concentration of Fe^3+^ and Fe^2+^ is quite low (5–30 *μ*M), which did not interfere with our detection system. In contrast, Na^+^, K^+^, Mg^2+^, and Ca^2+^ (500 *μ*M) induced fluorescence enhancement to a certain degree. Since Cu^2+^ demonstrated the largest fluorescence intensity quenching among these conventional metal ions, Cu^2+^ was employed to construct the on-off fluorescent system for PPase activity assay.

**Figure 4 fig4:**
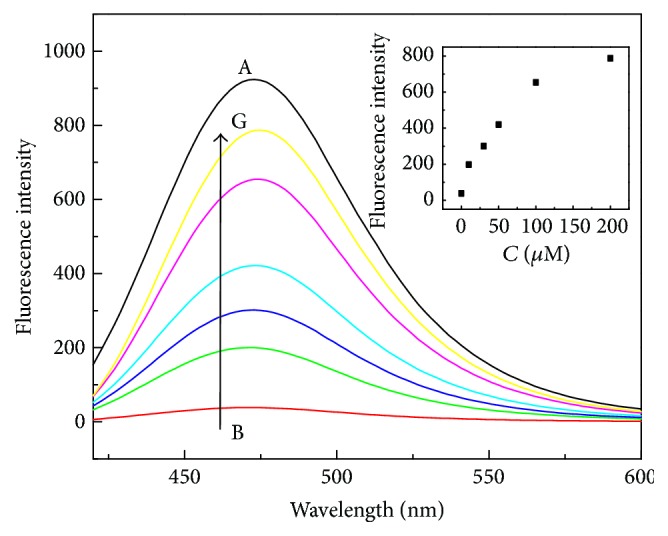
Fluorescence responses of DHC (500 *μ*M) (curve A) and Cu^2+^-DHC (Cu^2+^: 200 *μ*M, DHC: 500 *μ*M) complex with PPi (curves B to G: 0, 10, 30, 50, 100, and 200 *μ*M) in HEPES aqueous buffer (10 mM, pH 6.5). Inset shows fluorescence changes of Cu^2+^-DHC (Cu^2+^: 200 *μ*M, DHC: 500 *μ*M) complex at 475 nm as a function of PPi concentration. Other reaction conditions are the same as Figure 1.

To investigate the different concentration of PPi sensing in Cu^2+^-DHC complex system, fluorescence responses of Cu^2+^-DHC complex with different concentrations of PPi in HEPES buffer solutions (10 mM, pH 6.5) were recorded, as showed in Figure 4; it can be seen that, with the increasing concentration of PPi, the fluorescence emission intensity of DHC was restored gradually. This observation can be attributed to the fact that the coordination effect of PPi with Cu^2+^ is stronger than that of DHC with Cu^2+^ and DHC was released from Cu^2+^-DHC complex as demonstrated in Figure 1(a). The fluorescence emission intensity had the dependence on the concentrations of PPi in the range from 10 to 200 *μ*M with the detection limit of 580 nM, which fit well for the detection PPi in some biological fluids, such as plasma and serum in which PPi was found to be around the micromolar range. Therefore, these results demonstrated that the in situ formed Cu^2+^-DHC complex could be a promising “off-on” type fluorescence sensor for the PPi based on the displacement approach. Additionally, 200 *μ*M PPi can induce the fluorescence 90% recovery and thus was chosen in further assays.

**Figure 5:**
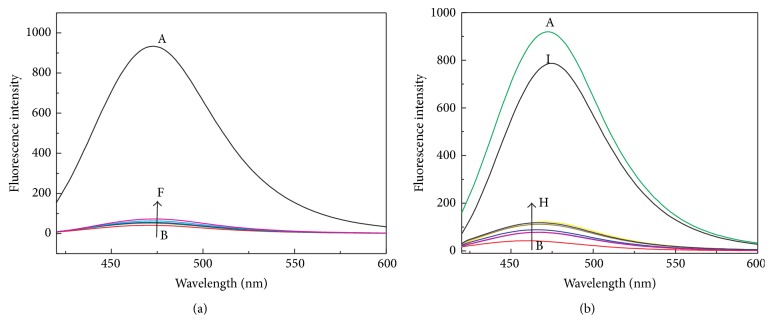
(a) Fluorescence responses of DHC (500 *μ*M) (curve A) and Cu^2+^-DHC (Cu^2+^: 200 *μ*M, DHC: 500 *μ*M) complex with HPO_4_^2−^ (curves B to F: 0, 10, 30, 50, 100, and 200 *μ*M) in HEPES aqueous buffer (10 mM, pH 6.5). (b) Fluorescence changes of DHC (500 *μ*M, curve A) and Cu^2+^-DHC (Cu^2+^: 200 *μ*M, DHC: 500 *μ*M) complex in the absence (curve B) and presence of various anions (curves C to I: PO_4_^3−^, HPO_4_^2−^, H_2_PO_4_^−^, AMP, ADP, ATP, and PPi, 200 *μ*M) in HEPES buffer aqueous solution (10 mM, pH 6.5). Other reaction conditions are the same as Figure 1.

To further examine the feasibility of the as-designed sensing method, the interference from the products produced from the PPase-based biocatalytic reaction was studied. The direct product of PPase-catalyzed hydrolysis of PPi is PO_4_^3−^, and the protonation states of PO_4_^3−^ including PO_4_^3−^, HPO_4_^2−^, and H_2_PO_4_^−^ are present in the different pH. According to acid dissociation constants of H_3_PO_4_ (pK_a1_ = 2.12, pK_a2_ = 7.20, and pK_a3_ = 12.36), in HEPES buffer (10 mM, pH 6.5), HPO_4_^2−^ (namely, Pi) exists dominantly. In our designed strategy, the addition of PPase to the Cu^2+^-PPi complex containing DHC obviously leads to the fluorescence quenching again because Pi, which is produced from PPase-catalyzed hydrolysis of PPi, cannot chelate Cu^2+^ to form the complex. Therefore, in order to define chelation interaction between Pi and Cu^2+^, fluorescence titration with Pi in varying concentrations was conducted in Cu^2+^-DHC complex system. As shown in Figure 5(a), with the increasing concentration of Pi, the fluorescence emission intensity of DHC was not restored gradually as well as PPi, suggesting no chelation interaction between Pi and Cu^2+^. Furthermore, all the phosphate-related anions, including PO_4_^3−^, HPO_4_^2−^, H_2_PO_4_^−^, AMP, ADP, and ATP, were also investigated in this study. As shown in Figure 5(b), all the phosphate-related anions except PPi restored the fluorescence intensity of DHC weakly, essentially demonstrating that these substances do not interfere with the PPase activity assay because of their weaker coordination capability with Cu^2+^. This feature actually forms the basis for the development of the effective fluorescence method for sensitive PPase activity assay, as demonstrated in Figures 2 [Fig fig3] [Fig fig4]–5.

### 3.3. Interference Study for PPi and PPase

**Figure 6:**
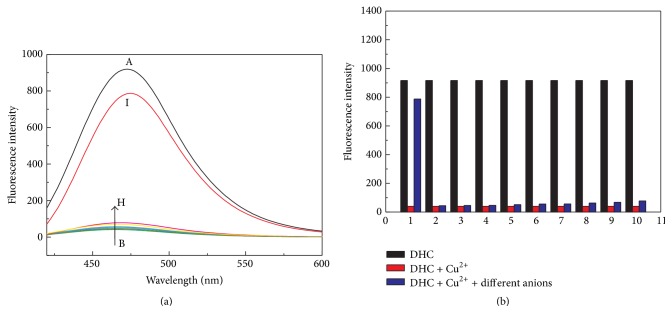
(a) Fluorescence changes of DHC (500 *μ*M, curve A) and Cu^2+^-DHC (Cu^2+^: 200 *μ*M, DHC: 500 *μ*M) complex in the absence (curve B) and presence of various anions (curves C to I: Cl^−^, F^−^, Br^−^, I^−^, SO_4_^2−^, ClO_4_^−^, NO_3_^−^, CH_3_CO_2_^−^, CO_3_^2−^, and PPi; 200 *μ*M) in HEPES buffer aqueous solution (10 mM, pH 6.5). (b) Fluorescence responses of DHC (500 *μ*M) (black bars) and Cu^2+^-DHC (Cu^2+^: 200 *μ*M, DHC: 500 *μ*M) complex at 475 nm in the absence (red bars) and presence of different anions (1 to 10: PPi, Cl^−^, F^−^, Br^−^, I^−^, SO_4_^2−^, ClO_4_^−^, NO_3_^−^, CH_3_CO_2_^−^, and CO_3_^2−^; 200 *μ*M) (blue bars). Other reaction conditions are the same as Figure 1.

**Figure 7:**
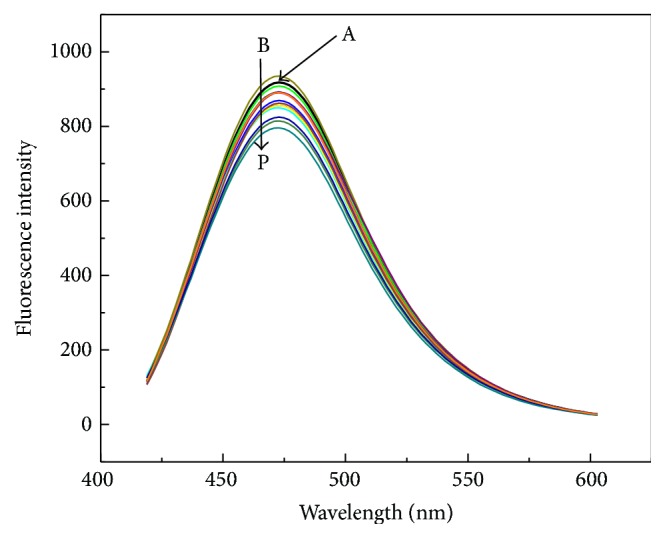
Fluorescence changes of DHC (500 *μ*M, curve A) in the absence (curve A) and presence of various anions (curves B to P: Cl^−^, F^−^, Br^−^, I^−^, SO_4_^2−^, ClO_4_^−^, NO_3_^−^, CH_3_CO_2_^−^, CO_3_^2−^, ATP, AMP, ADP, PPi, PO_4_^3−^, HPO_4_^2−^, H_2_PO_4_^−^, and PPi, 200 *μ*M) in HEPES buffer aqueous solution (10 mM, pH 6.5). Other reaction conditions are the same as Figure 1.

The selectivity of the as-designed sensing method for PPi was also evaluated by comparing the fluorescence response toward PPi (200 *μ*M) with 9 other kinds of anions, including Cl^−^, F^−^, Br^−^, I^−^, SO_4_^2−^, ClO_4_^−^, NO_3_^−^, CH_3_CO_2_^−^, and CO_3_^2−^ (200 *μ*M) in HEPES buffer aqueous solution (10 mM, pH 6.5) containing 500 *μ*M DHC and 200 *μ*M Cu^2+^ under the same detection condition as described in Section 3.2. As shown in Figure 6(a), the results demonstrated that these anions caused minor fluorescence intensity changes of Cu^2+^-DHC complex at 475 nm (the blue columns in Figure 6(b)) while PPi restored about 90% fluorescence intensity of DHC. Thus, the in situ generated Cu^2+^-DHC complex can behave as a high selective fluorescence “off-on” sensor for PPi. Additionally, the selectivity of DHC for sensing Cu^2+^ was evaluated by comparing its fluorescence response toward Cu^2+^ with those of 17 other kinds of conventional anions including Cl^−^, F^−^, Br^−^, I^−^, SO_4_^2−^, ClO_4_^−^, NO_3_^−^, CH_3_CO_2_^−^, CO_3_^2−^, ATP, AMP, ADP, PPi, PO_4_^3−^, HPO_4_^2−^, H_2_PO_4_^−^, and PPi (200 *μ*M), as shown in Figure 7. The results demonstrated that these tested anions induced negligible fluorescence intensity changes of DHC. Therefore, the proposed strategy has a high selectivity for PPi, allowing potential applications in complex samples.

Since the proposed fluorescent system was to be applied for the measurements of PPi and PPase in biological samples, it was necessary to validate whether there were interferences on this sensing system from ammonium ion, EDTA, and sulfhydryl compounds including GSH, Cys, and Hcy, all of which can coordinate with Cu^2+^. As shown in Figures  S-2 and S-3 (see Supplementary Material available online at http://dx.doi.org/10.1155/2016/4306838), the results showed that five substances as interferences had less observable fluorescent responses and can restore the fluorescence emission intensity of DHC compared with PPi. On the other hand, EDTA does not exist in body fluids, suggesting that EDTA had no interference on this fluorescent sensing system for the application in biological samples. Furthermore, the above-mentioned physiological substances, including NH_4_^+^, GSH, Cys, and Hcy, keep at the low concentrations, which do not exceed 50 *μ*M in biological samples. In such the concentration range, these four physiological substances had the slight fluorescent responses, indicating that these substances within a certain range of their concentration related to physiological changes have no interference on this fluorescent sensing system for the application in biological samples.

### 3.4. Quantitative Monitoring of PPase Activity

**Figure 8:**
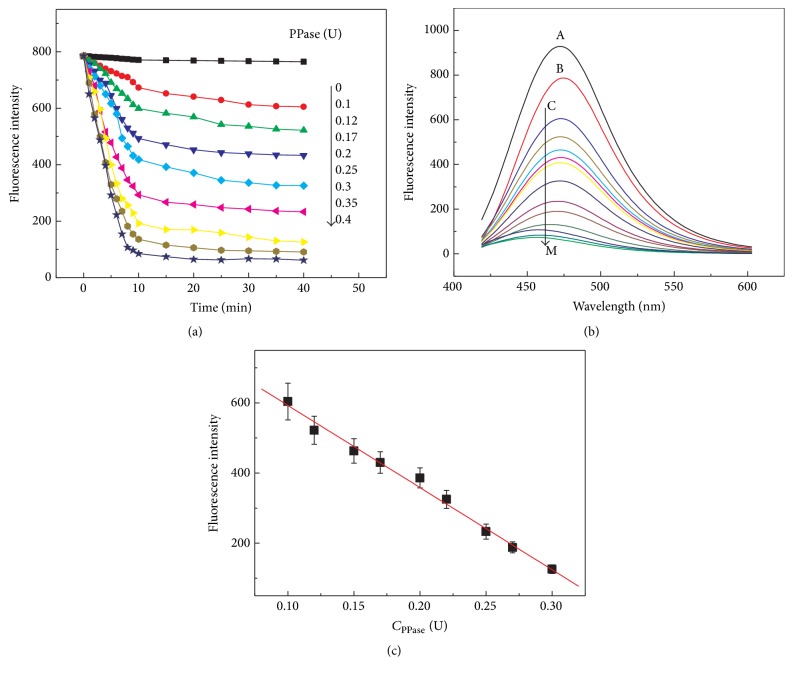
(a) Kinetic plots of time-dependent fluorescence responses versus different activities of PPase present. (b) Fluorescence responses of DHC (500 *μ*M) (curve A) and Cu^2+^-PPi (Cu^2+^: 200 *μ*M, PPi: 200 *μ*M) complex with DHC (500 *μ*M) were recorded at a time point of 30 min after the addition of PPase (curves B to M: 0, 0.1, 0.12, 0.15, 0.17, 0.2, 0.22, 0.25, 0.27, 0.3, 0.35, and 0.4 U) in HEPES aqueous buffer (10 mM, pH 6.5) in the presence of 500 *μ*M Mg^2+^. The final volume of each resulting mixture was adjusted to 500 *μ*L. (c) Fluorescence responses versus *C*_PPase_. Error bars were estimated from three replicate measurements. Other reaction conditions are the same as Figure 1.

It was reported that PPase is highly active in catalyzing the cleavage reaction of PPi to Pi in the presence of Mg^2+^ (log⁡*K*_Mg-PPi_ = 5.6 [30]). Herein, we attempted to apply the as-designed turn off-on-off fluorescent system for PPase activity assay in the presence of 500 *μ*M Mg^2+^. Typically, PPase with various concentrations was added into HEPES buffer aqueous solution containing 500 *μ*M DHC, 200 *μ*M Cu^2+^, 200 *μ*M PPi, and 500 *μ*M Mg^2+^.  Figure 8(a) depicts the typical time-dependent fluorescence spectra of the detection system. The spectra were consecutively recorded every 1 min at 25°C immediately after the addition of PPase to this system in the first 10 minutes and every 5 min in the later 30 minutes. For comparison, the time-dependent fluorescence spectrum of the detection system without the addition of PPase was constant (Figure 8(a), black curve). The interaction time required for optimum fluorescence assay in the presence of PPase was investigated in Figure 8(a). It is clearly seen that the emission peak at 475 nm sharply decreases from 0 to 10 min and then slightly decreases and finally keeps a plateau after 30 min. This suggests that PPase-catalyzed hydrolysis of PPi has finished within 30 min. Therefore, fluorescence responses were recorded at a time point of 30 min after the addition of PPase.

As typically displayed in Figure 8(b), fluorescence responses decrease with increasing PPase activity from 0.1 to 0.4 U (the final volume 500 *μ*L).  Figure 8(c) shows the fluorescence intensity at 475 nm versus the PPase concentrations. The linear equation was *F* = 827.09 − 2342.84*C*_PPase_ (U/500 *μ*L) (*R* ^2^ = 0.996) in the range from 0.1 to 0.3 U. The detection limit of PPase based on a signal-to-noise ratio of 3 was calculated to be 0.028 U in 500 *μ*L. These results demonstrated that our system was comparable and even more efficient for detection of the activity of PPase compared to those provided by other approaches [13, 14, 23]. Moreover, the selectivity of our method for PPase was verified by, respectively, testing some nonspecific proteins including BSA, egg protein, IgG, and PSA with different isoelectric points and structures (figure has not been shown). The result demonstrated that no remarkable fluorescence changes were induced by these tested nonspecific proteins, suggesting that the proposed strategy has a high selectivity in a quantitative PPase activity and allows potential applications in complex samples.

In order to evaluate the validity of the developed method, especially on interference arising from DHC and Cu^2+^ for PPase activity assay, a colorimetric method using molybdenum blue for PPase activity assay [29] was performed as described in Section 2.4. The results in a microplate reader showed that the absorbance at 650 nm was 0.732, 0.716, and 0.789 in the absence and the presence of 200 *μ*M Cu^2+^ or 500 *μ*M DHC, respectively. This suggests that the presence of 200 *μ*M Cu^2+^ or 500 *μ*M DHC did not affect the PPase activity. The above results of the microplate reader are also according to UV-Vis absorption responses and photos perfectly (see Figure  S-1).

### 3.5. Inhibitor Efficiency Evaluation

**Figure 9:**
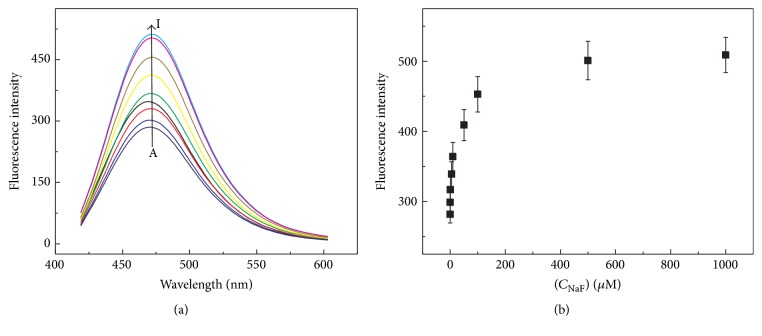
(a) Fluorescence responses of Cu^2+^-PPi (Cu^2+^: 200 *μ*M, PPi: 200 *μ*M) complex with DHC (500 *μ*M) after adding different concentration of NaF-treated PPase (0.25 U) in HEPES aqueous buffer (10 mM, pH 6.5). The concentration of NaF (curves A to I: 0, 0.1, 1, 5, 10, 50, 100, 500, and 1000 *μ*M). The final volume of each resulting mixture was adjusted to 500 *μ*L. (b) Fluorescence intensity versus the NaF concentration. Error bars were estimated from three replicate measurements. Other reaction conditions are the same as Figure 1.

Sodium fluoride (NaF) has been reported to be an inhibitor of PPase activity through the formation of the [F−PPase] intermediate [31]. Here, NaF as a model inhibitor was investigated using the developed method in this study. We first studied whether NaF could induce the fluorescence intensity changes of DHC or Cu-DHC complex. Comparing Figure 9 with Figures 6 and 7, the sole addition of NaF to DHC or Cu^2+^-DHC complex caused negligible fluorescence intensity changes. This observation indicates that NaF itself could not interfere with fluorescence emission in both these sensing systems. The inhibitor efficiency evaluation was performed as described in Section 2.5. The fluorescence responses at 475 nm were restored gradually with the increasing NaF concentration from 0.1 to 1000 *μ*M, as showed in Figure 9. This indicates that NaF could remarkably inhibit the PPase activity.

To further confirm that the fluorescence recovery resulted from the NaF-inhibited PPase, we also checked the inhibition effect of NaF on the fluorescence system containing Cu^2+^ (200 *μ*M) and PPi (200 *μ*M) and DHC (500 *μ*M). The results showed that 2 mM NaF could not affect the fluorescence emission of the Cu^2+^-PPi-DHC system (figure has not been shown). This observation suggests that the as-developed fluorescence method in this study can be potentially used to evaluate the inhibitor efficiency of NaF for PPase activity.

It has been reported that PPase activity could be inhibited by micromolar F^−^ ion [31]. Herein, we found that the restored fluorescence emission of the tested system reached constant when 0.25 U of PPase (500 *μ*L) was preincubated with 500 *μ*M NaF, and the higher concentration of NaF (1000 *μ*M) cannot restore the fluorescence intensity again. These observations suggest that the concentration of NaF less than 500 *μ*M could remarkably inhibit the activity of 0.25 U of PPase. Moreover, in order to evaluate the inhibition effect of NaF, IC_50_, which represents the concentration of an inhibitor that is required for 50% inhibition of an enzyme, could be used as a parameter to evaluate the inhibitors. As can be seen from Figure 9(b), a profile was obtained by plotting the fluorescence intensity versus the NaF concentration. The IC_50_ value of PPase was calculated to be about 18.2 ± 2.0 *μ*M. These results essentially demonstrated that the turn off-on-off fluorescent system could also be used to evaluate the inhibitor efficiency and may further be applied to screen other PPase inhibitors simply.

## 4. Conclusion

We have designed and constructed a simple and efficient turn off-on-off fluorescent system based on PPase-regulated competitive coordination of Cu^2+^ between a water-soluble fluorescent probe DHC and inorganic ion PPi, which shows a highly selective and sensitive fluorescence response toward PPi and PPase over other tested anions and structural analogues in a 100% aqueous medium. The developed method with good linearity and low detection limit bears advantages in the low technical and instrumental demands and could be also very promising for PPase inhibitor efficiency evaluation. Thus, the present turn off-on-off fluorescent strategy holds great potential for applications in the reliable clinic sensing of PPi in biological and environmental samples and the PPase-related mechanism research and disease diagnostics.

## Supplementary Material


Figure S1: UV-Vis absorption responses of PPase activity assays based on colorimetric method of molybdenum blue in the absence (red line) and the presence of 200 *μ*M (blue line) or 500 *μ*M DHC (black line), respectively. The reaction conditions are shown in 2.4 of the experimental section. Inset: the corresponding photos of PPase activity assays based on colorimetric method of molybdenum blue in the absence (left) and the presence of 200 *μ*M Cu^2+^ (middle) or 500 *μ*M DHC (right).
Figure S2: (A) Fluorescence responses of DHC (500 *μ*M) (curve a), Cu^2+^(200 *μ*M) - DHC (500 *μ*M) complex with NH_3_Cl (curve b to j: 0, 10, 30, 50, 100, 200, 400, 600, 800 *μ*M) in HEPES aqueous buffer (10 mM, pH 6.5). (B) Fluorescence responses of DHC (500 *μ*M) (curve a), Cu^2+^(200 *μ*M) - DHC (500 *μ*M) complex with EDTA (curve b to j: 0, 5, 10, 30, 50, 70, 100, 200, 400 *μ*M) in HEPES aqueous buffer (10 mM, pH 6.5). Other reaction conditions are the same as Figure 1.
Figure S3: Fluorescence responses of DHC (500 *μ*M) (curve a), Cu^2+^(200 *μ*M) - DHC (500 *μ*M) complex with GSH (A, curve b to g: 0, 30, 50, 100, 200, 400 *μ*M), Cys (B, curve b to i: 0, 10, 30, 50, 100, 200, 400, 800 *μ*M), Hcy (C, curve b to j: 0, 10, 30, 50, 100, 200, 400, 800 *μ*M) in HEPES aqueous buffer (10 mM, pH 6.5). Other reaction conditions are the same as Figure 1.
