# Supplementary material for: Construction of a Turn Off-On-Off Fluorescent System Based on Competitive Coordination of Cu2+ between 6,7-Dihydroxycoumarin and Pyrophosphate Ion for Sensitive Assay of Pyrophosphatase Activity
Source: J Anal Methods Chem. 2016 Sep 27;2016:4306838. doi: 10.1155/2016/4306838 (PMC5059578; doi:10.1155/2016/4306838)
Supplement: Supplementary file 1 — Figure S1: UV-Vis absorption responses of PPase activity assays based on colorimetric method of molybdenum blue in the absence (red line) and the presence of 200 μM (blue line) or 500 μM DHC (black line), respectively. The reaction conditions are shown in 2.4 of the experimental section. Inset: the corresponding photos of PPase activity assays based on colorimetric method of molybdenum blue in the absence (left) and the presence of 200 μM Cu2+ (middle) or 500 μM DHC (right). Figure S2: (A) Fluorescence responses of DHC (500 μM) (curve a), Cu2+(200 μM) - DHC (500 μM) complex with NH3Cl (curve b to j: 0, 10, 30, 50, 100, 200, 400, 600, 800 μM) in HEPES aqueous buffer (10 mM, pH 6.5). (B) Fluorescence responses of DHC (500 μM) (curve a), Cu2+(200 μM) - DHC (500 μM) complex with EDTA (curve b to j: 0, 5, 10, 30, 50, 70, 100, 200, 400 μM) in HEPES aqueous buffer (10 mM, pH 6.5). Other reaction conditions are the same as Figure 1. Figure S3: Fluorescence responses of DHC (500 μM) (curve a), Cu2+(200 μM) - DHC (500 μM) complex with GSH (A, curve b to g: 0, 30, 50, 100, 200, 400 μM), Cys (B, curve b to i: 0, 10, 30, 50, 100, 200, 400, 800 μM), Hcy (C, curve b to j: 0, 10, 30, 50, 100, 200, 400, 800 μM) in HEPES aqueous buffer (10 mM, pH 6.5). Other reaction conditions are the same as Figure 1. [file 4306838.f1.doc]

***Supporting Information***

**Construction of a Turn Off-On-Off Fluorescent System Based on Competitive Coordination of Cu^2+^ between 6, 7-dihydroxycoumarin and Pyrophosphate Ion**

**For Sensitive Assay of Pyrophosphatase Activity**

Lingzhi Zhao^a,b^, Liu Zhao^c^, Yanqing Miao^a^, Chunye Liu ^a^, Chenxiao Zhang^b^

a Department of pharmacy, Xi 'an Medical College, Xi 'an 710021, China

b Laboratory of Analytical Chemistry for Life Science of Shaanxi Province, School of Chemistry and Chemical Engineering, Shaanxi Normal University, Xi’an 710062, China

c Beijing Research Center of Agricultural Standards and Testing, Beijing 100097,

Key Corresponding Author. E-mail: oldskyhappy_zlz@163.com


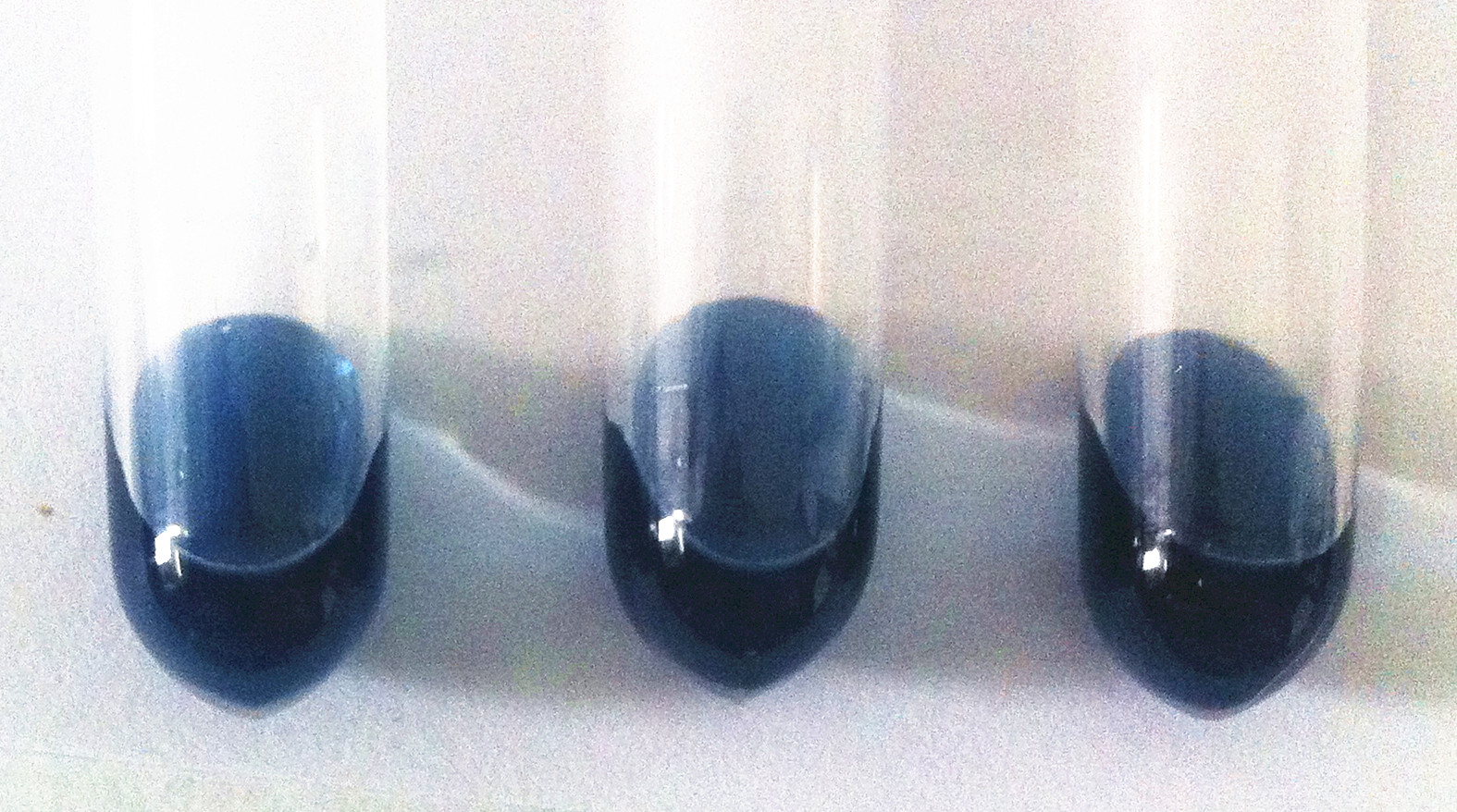

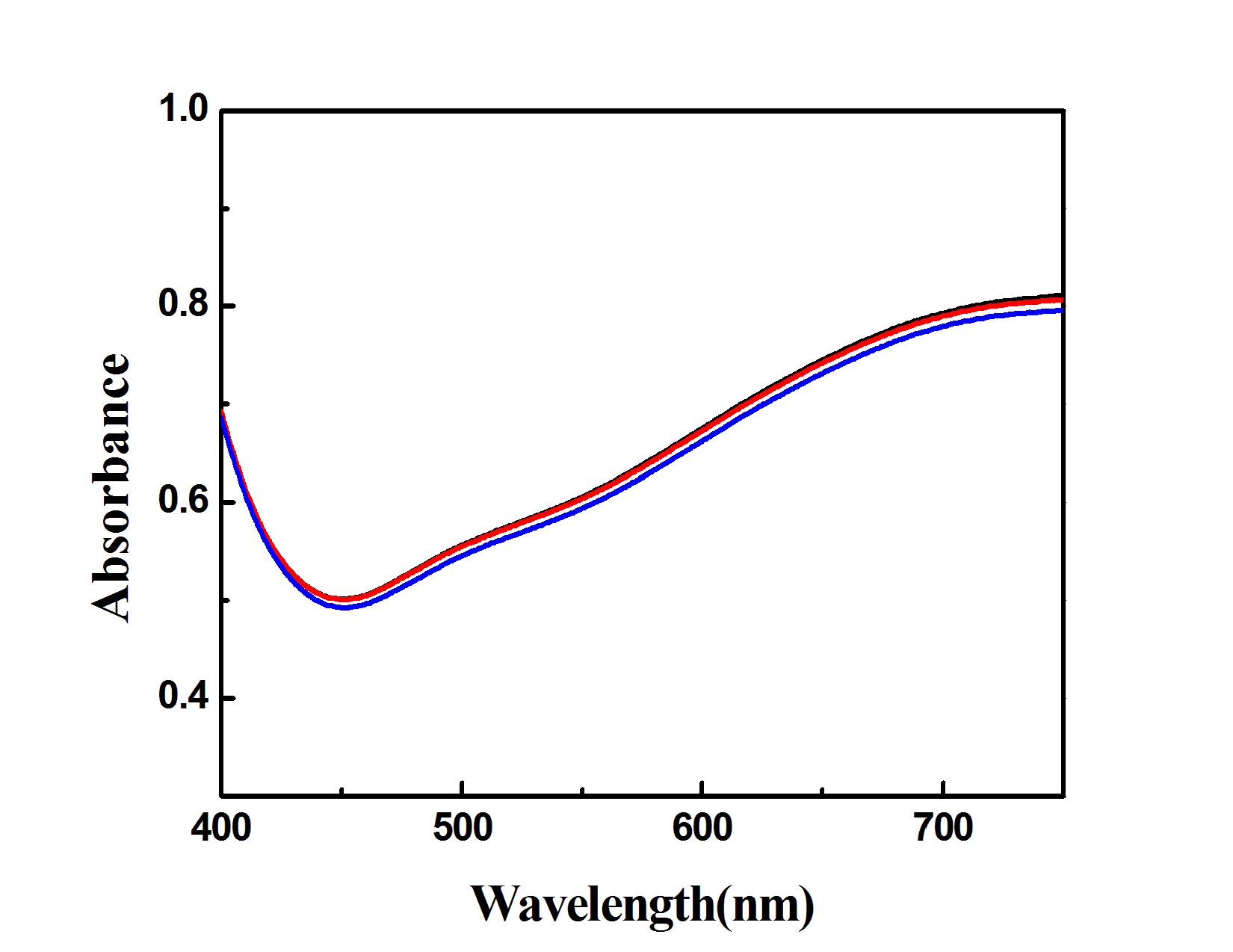


**Fig. S-1** UV-Vis absorption responses of PPase activity assays based on colorimetric method of molybdenum blue in the absence (red line) and the presence of 200 μM (blue line) or 500 μM DHC (black line), respectively. The reaction conditions are shown in 2.4 of the experimental section. Inset: the corresponding photos of PPase activity assays based on colorimetric method of molybdenum blue in the absence (left) and the presence of 200 μM Cu^2+^ (middle) or 500 μM DHC (right)


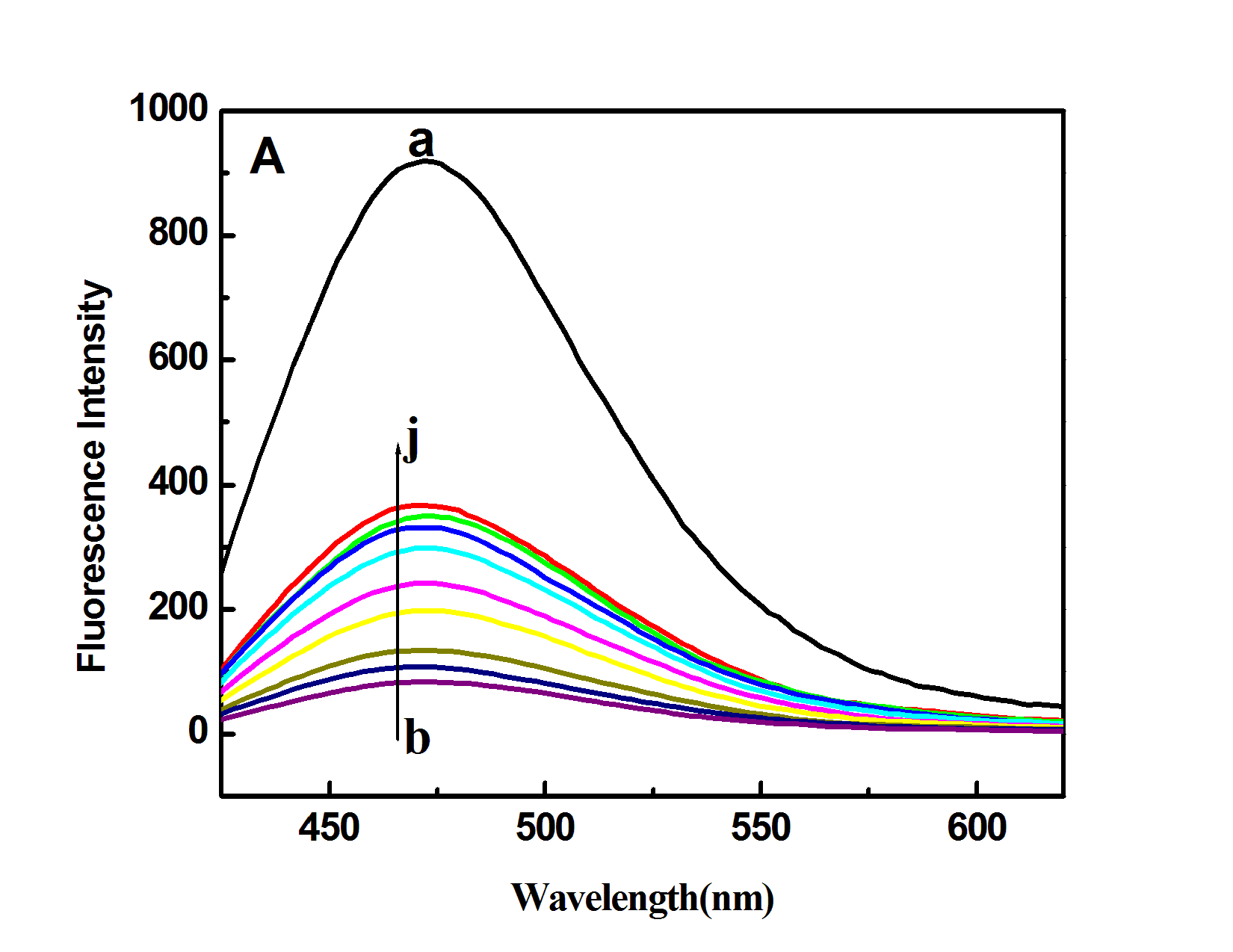

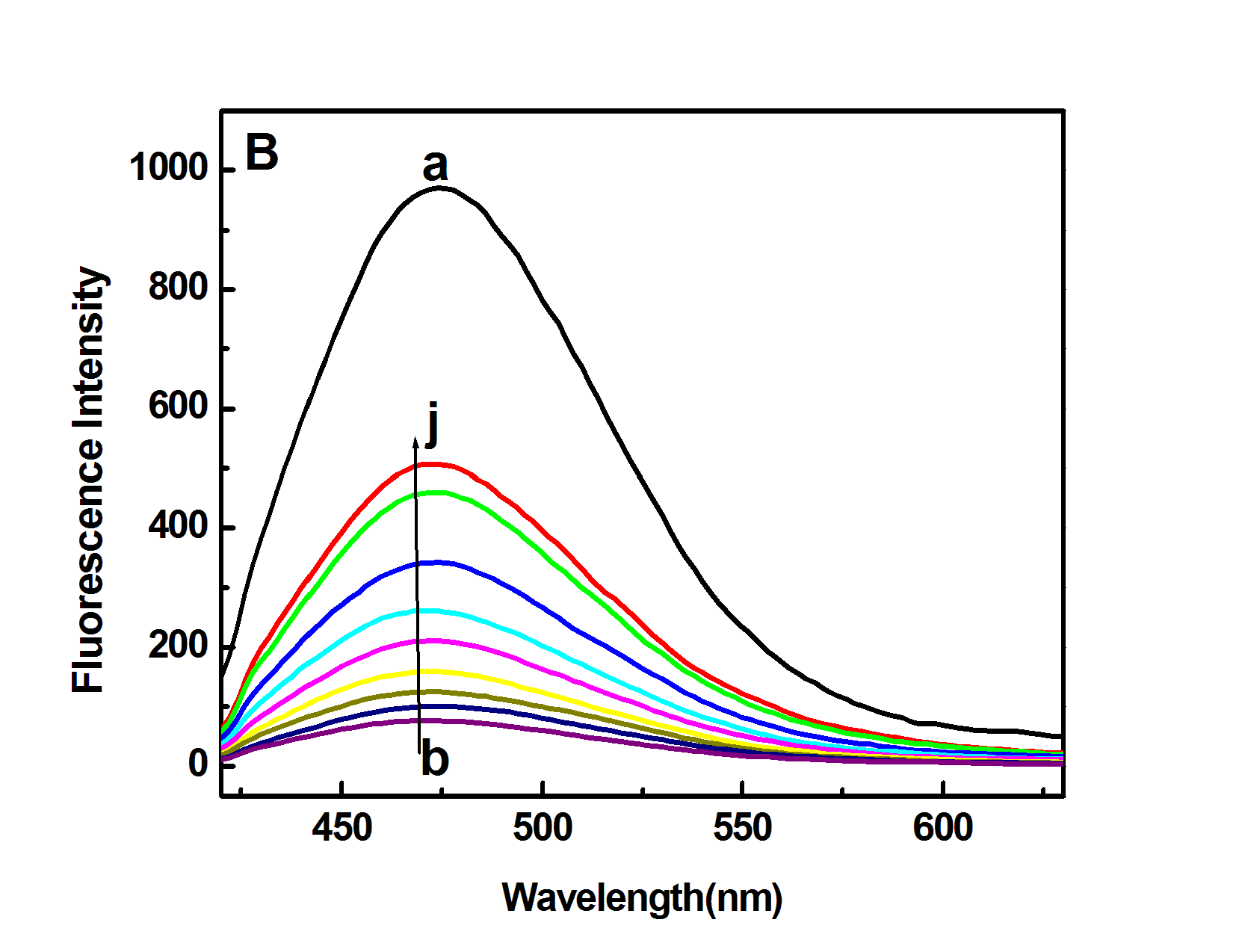


**Fig. S-2.** (A) Fluorescence responses of DHC (500 μM) (curve a), Cu^2+^(200 μM) - DHC (500 μM) complex with NH_3_Cl (curve b to j: 0, 10, 30, 50, 100, 200, 400, 600, 800 μM) in HEPES aqueous buffer (10 mM, pH 6.5). (B) Fluorescence responses of DHC (500 μM) (curve a), Cu^2+^ (200 μM) - DHC (500 μM) complex with EDTA (curve b to j: 0, 5, 10, 30, 50, 70, 100, 200, 400 μM) in HEPES aqueous buffer (10 mM, pH 6.5). Other reaction conditions are the same as FIGURE 1.


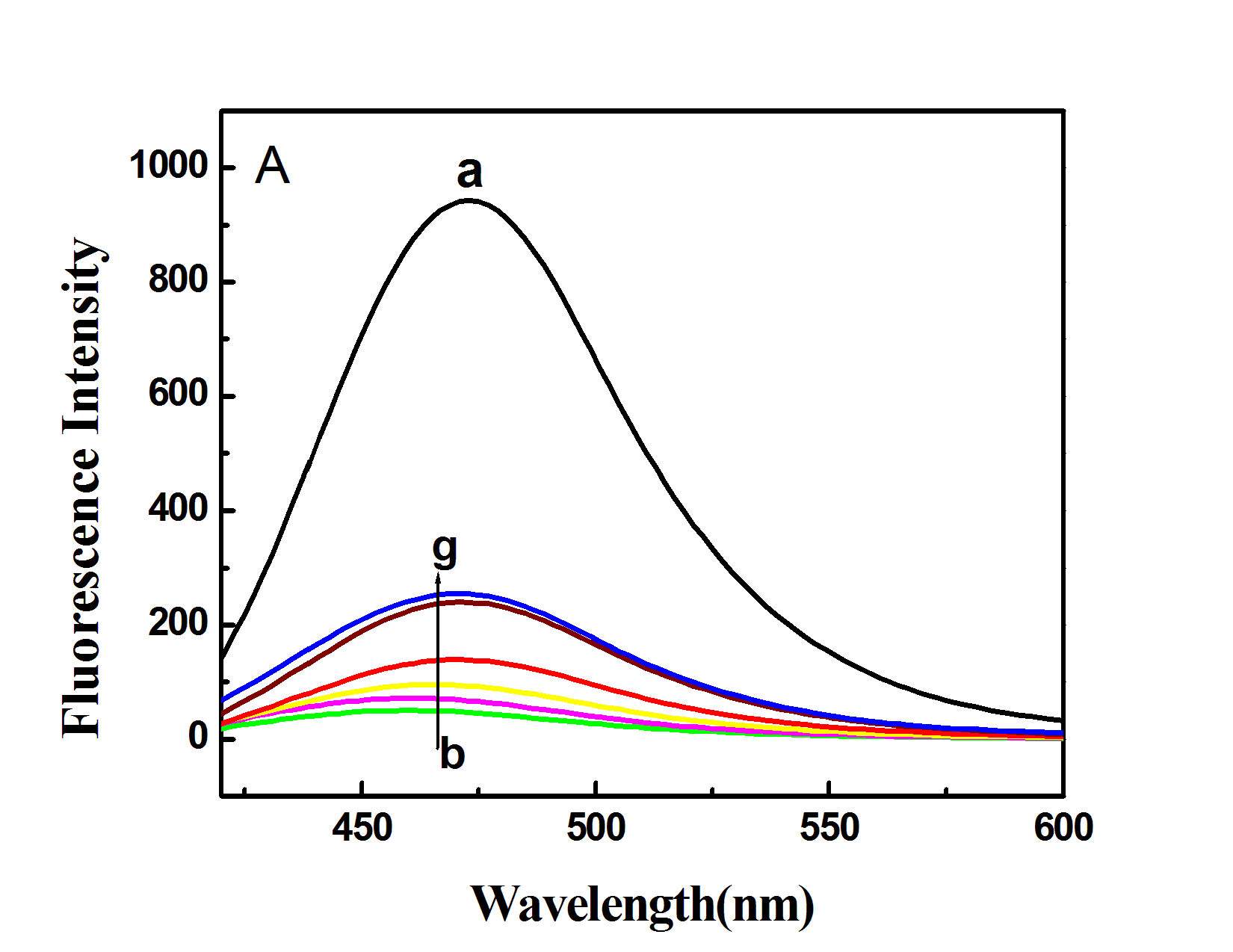

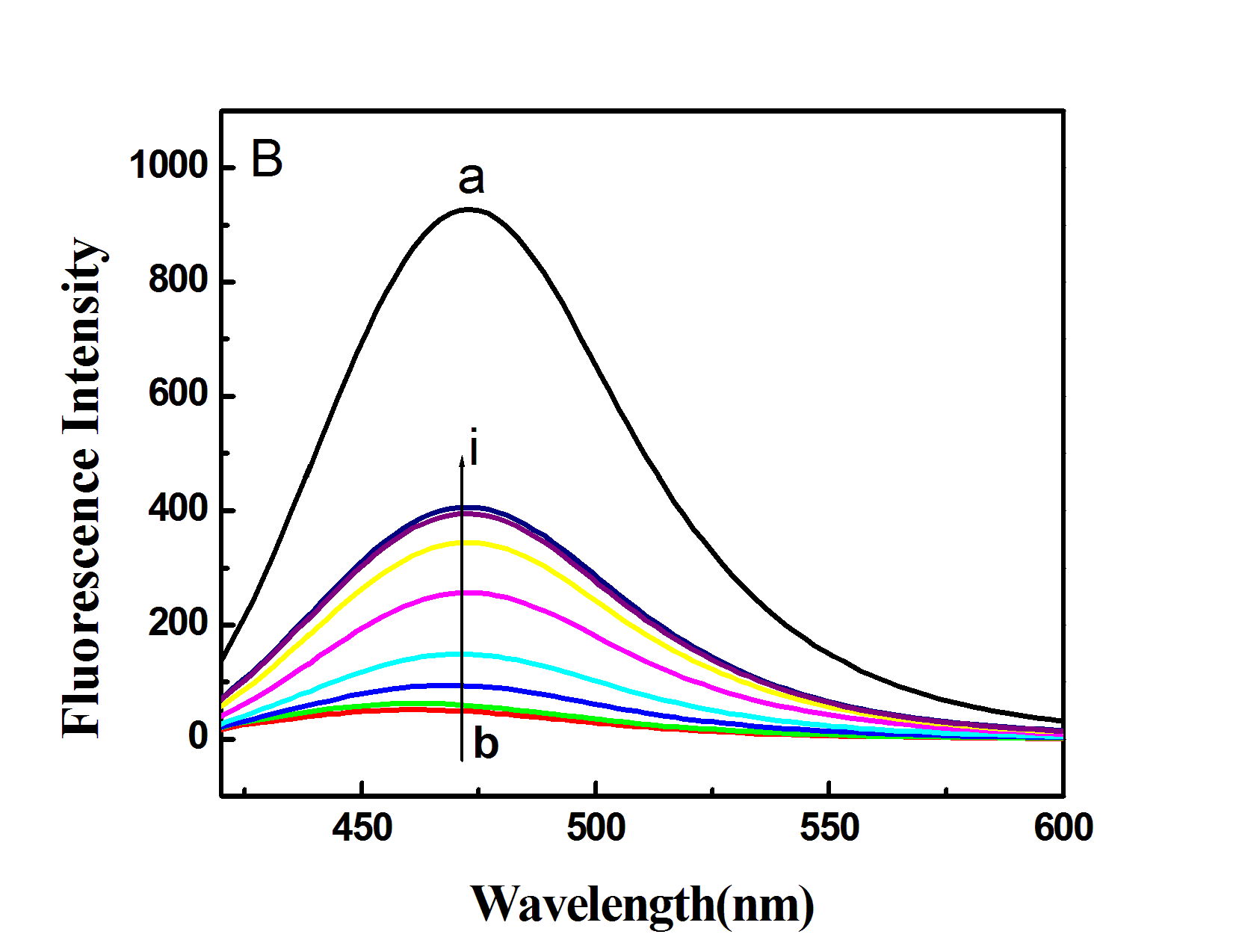


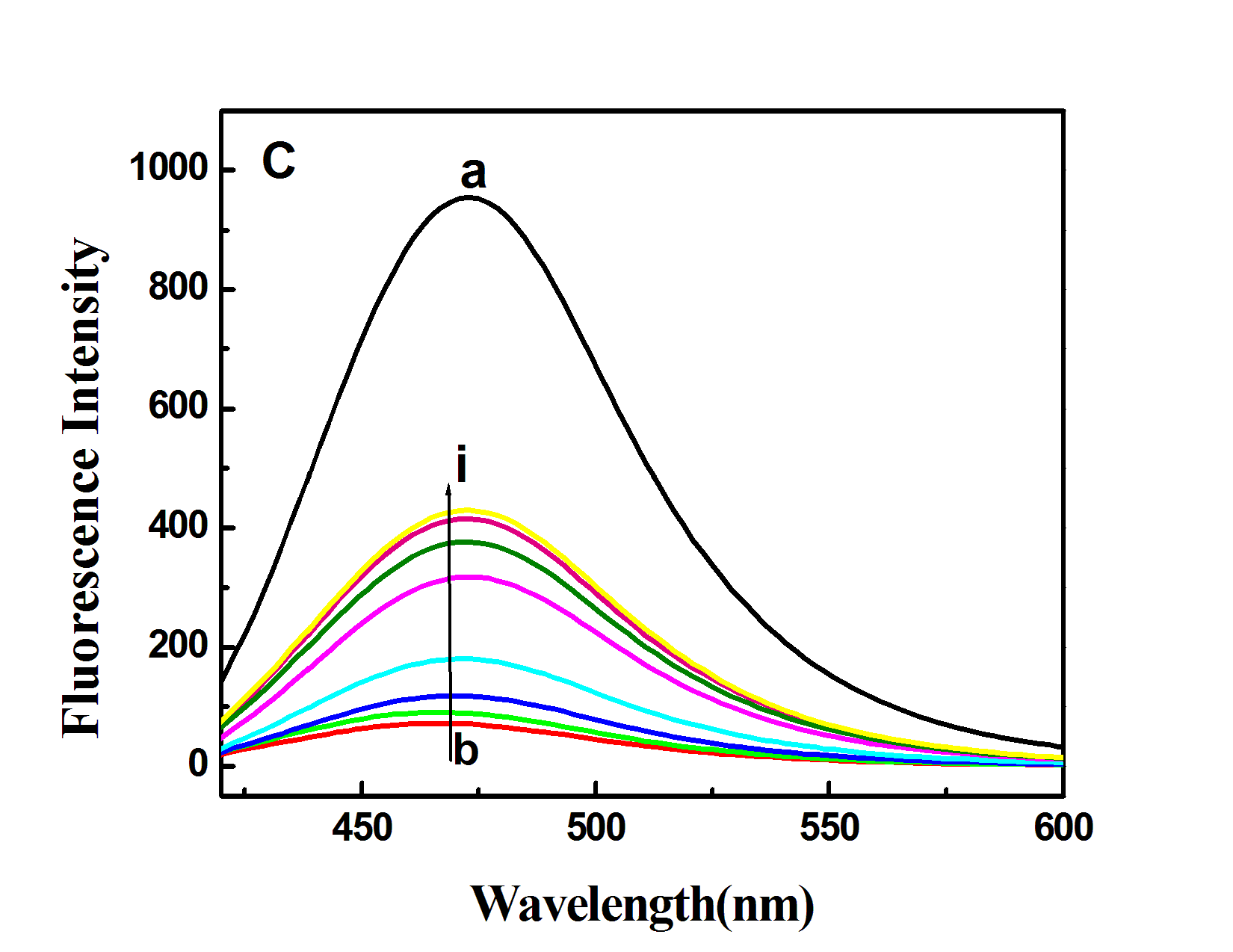


**Fig. S-3.** Fluorescence responses of DHC (500 μM) (curve a), Cu^2+^(200 μM) - DHC (500 μM) complex with GSH (A, curve b to g: 0, 30, 50, 100, 200, 400 μM), Cys (B, curve b to i: 0, 10, 30, 50, 100, 200, 400, 800 μM), Hcy (C, curve b to j: 0, 10, 30, 50, 100, 200, 400, 800 μM) in HEPES aqueous buffer (10 mM, pH 6.5). Other reaction conditions are the same as FIGURE 1.
